# Transcriptional activity of the long control region in human papillomavirus type 33 intratype variants

**DOI:** 10.1186/s12985-023-02114-y

**Published:** 2023-07-17

**Authors:** Eszter Gyöngyösi, Brigitta László, Anita Szalmás, József Kónya, György Veress

**Affiliations:** Department of Medical Microbiology, Faculty of Medicine, University of Debrecen, Nagyerdei krt. 98, Debrecen, H-4032 Hungary

**Keywords:** Human papillomavirus, Type 33, Long control region, Variants, Transcriptional activity

## Abstract

**Background:**

High-risk human papillomaviruses (HPVs) are responsible for the development of cervical and other anogenital cancers. Intratype sequence variants of certain high-risk HPV types (e.g. 16, 18 and 31) are thought to have different oncogenic potential, partly due to nucleotide sequence variation in the viral long control region (LCR). The LCR has an important role in the regulation of viral replication and transcription. The purpose of this study was to explore sequence variation in the LCR of HPV 33 intratype variants in Hungary and to see whether there are differences in the transcriptional activities of the variants.

**Methods:**

The complete HPV 33 LCR was amplified from HPV 33 positive cervical samples. After sequencing the LCR variants, multiple sequence alignment and phylogenetic analyses were carried out. Representative HPV 33 LCR sequence variants were selected for cloning and functional analysis. After transient transfection of HeLa cells, luciferase reporter assays were used to analyse the transcriptional activities of different LCR variants.

**Results:**

Altogether 10 different variants were identified by sequence analysis of the HPV 33 LCR. The results of phylogenetic analysis showed that 3 variants belonged to sublineage A1, while the other 7 variants clustered with sublineage A2. Variants belonging to sublineage A2 had significantly lower transcriptional activities than variants belonging to sublineage A1. Within sublineage A2, the two variants analysed had significantly different transcriptional activities, which was shown to be caused by the A7879G variation.

**Conclusions:**

Nucleotide variation in the HPV 33 LCR can result in altered transcriptional activity of the intratype variants. Our results can help to understand the correlation between LCR polymorphism and the oncogenic potential of HPV 33 variants.

## Background

High-risk human papillomaviruses (HPV), included in the *Alphapapillomavirus* genus of the *Papillomaviridae* family, are responsible for the development of cervical cancer and some other anogenital or head and neck cancers [1, 2]. Within the *Alphapapillomavirus* genus, the types most frequently causing cervical cancer are HPV 16, 18, 31, 33, 45, 52 and 58 [3, 4]. These high-risk HPV types are also included in the nonavalent prophylactic HPV vaccine, along with the low-risk types HPV 6 and 11 [5].

The HPV genome is composed of three functional regions [6]. The early region encodes proteins (E1, E2, E4, E5, E6 and E7) that are mainly involved in the replication and transcription of the viral genome. In high-risk HPV types, the E6 and E7 proteins are called oncoproteins, because they are also involved in the transformation of host cells [6]. The late region contains the genes encoding the L1 and L2 capsid proteins. The long control region (LCR) contains regulatory elements that are involved in the replication of the viral genome and also in the transcription of the viral genes, including the E6 and E7 oncogenes [7].

Different HPV types have at least 10% difference in the nucleotide sequence of the L1 gene [8]. Within HPV types, isolates with less than 10% sequence divergence from each other are called intratype variants [8, 9]. There may be significant differences between intratype variants in clinical behaviour and also in biological characteristics [9–11].

HPV 33, the subject of this study, belongs to the species Alpha-9 of the *Alphapapillomavirus* genus, along with other high-risk types (HPV 16, 31, 35, 52, 58 and 67). HPV 33 DNA can be demonstrated in 4–5% of invasive cervical cancer cases worldwide [3, 4]. Intratype sequence variation was also studied in the case of HPV 33 [12–17]. Phylogenetic analysis of the variants revealed the presence of variant lineages A (containing sublineages A1, A2 and A3), B and C [12, 13]. The different lineages and sublineages were found to have characteristic geographic distribution [12]. In Europe, only sublineages A1 and A2 were demonstrated, while lineages B and C were found only in Africa. The rare sublineage A3 was exclusively found in Asia/Oceania [12].

The results of clinical studies suggest that there may be differences in the clinical behaviour (such as persistence and oncogenic potential) of different HPV 33 variants [11, 12, 18–23]. In vitro studies targeting different genomic regions of the virus revealed differences in the functional characteristics of HPV 33 intratype variants [24–31]. Some of these biological differences are probably responsible for the observed differences in the clinical behaviour of HPV 33 variants. Thus, it seems to be promising to proceed with the phylogenetic analysis and in vitro functional characterization of HPV 33 intratype variants.

The purpose of this study was to explore sequence variation in the LCR of HPV 33 intratype variants in Hungary and to see whether there are differences in the transcriptional activities of the variants.

## Methods

### Sample collection and HPV genotyping

Exfoliated cell specimens were collected from the uterine cervix of women with cytological and/or colposcopical abnormalities at the Department of Obstetrics and Gynaecology, Clinical Center, University of Debrecen, between 2008 and 2016. The mean age of women included in this study was 40 (25–73) years. Isolation of viral DNA and HPV genotyping were performed as recently described [32]. Twenty samples from 19 women found to be positive for HPV 33 were selected for sequence analysis of the LCR.

### Polymerase chain reaction (PCR)

The complete LCR of HPV 33 (nt 7089 − 126) was amplified using the primers 33LCR129 (5’-GCGCGGTACCAATAACACTTTGTGTAATTGTG-3’, nt 7089–7110) and 33LCR1075 (5’-GCGCAAGCTTCTCAGTGTCTTGAAACATAGTC-3’, nt 105–126) containing restriction enzyme recognition sites for *KpnI* and *HindIII* (underlined). PCR reactions were carried out with the GeneAmp High Fidelity PCR System (Applied Biosystems, Waltham, Massachusetts, USA) as previously described [32]. PCR amplimers were purified using Qiaquick Gel Extraction Kit (Qiagen, Hilden, Germany). Sequencing of purified PCR products was carried out by UD-GenoMed Medical Genomic Technologies Ltd. (Debrecen, Hungary) using Big Dye Terminator v3.1 Cycle Sequencing Kit (Applied Biosystems) and ABI Prism 3100-Avant Genetic Analyzer (Applied Biosystems).

### Cloning

Representative HPV 33 LCR sequence variants were directionally cloned into the promoterless luciferase reporter vector pGL2-Basic carrying the firefly (*Photinus pyralis*) luciferase gene (Promega, Madison, Wisconsin, USA), as previously described [32]. Incubation of bacteria after transformation was performed at 25 °C for 48 h (instead of 37 °C for 16 h) to reduce the toxicity of the HPV 33 LCR sequence on the host bacteria [32].

To prepare constructs containing deletions in the HPV 33 LCR, the same PCR conditions and cloning methods were used as described above, except for the primer pairs. To prepare the 33LCRD2 constructs, primers 33LCR547 (5’-GCGCGGTACCTTTCGGTTACTTGGCATACATA-3’, nt. 7507–7528) and 33LCR1075 were used. To prepare the 33LCRD3 constructs, the primers 33LCR846 (5’-GCGCGGTACCTATGCCAAACTATGCCTTGTAA-3’ nt. 7806–7827) and 33LCR1075 were used. The templates used in the PCR were the constructs containing the full-length HPV 33 LCR sequences. All plasmid constructs (containing different regions of different HPV 33 LCR variants) were verified by sequencing.

### Transient transfection and luciferase assay

Luciferase reporter assays were used for functional analysis of different HPV 33 LCR sequence variants. Lipofectamine 2000 Transfection Reagent (Thermo Fisher Scientific, Waltham, Massachussets, USA) was used to tranfect HeLa cells (a HPV 18 positive cervix carcinoma cell line). Hela cells were maintained in Dulbecco’s modified Eagle’s medium (DMEM, low glucose, pyruvate; Thermo Fisher Scientific) supplemented with 10% fetal calf serum, 2 mM L-glutamine and antibiotics (100 U/ml penicillin and 100 mg/ml streptomycin). Before transfection, cells were seeded on 6-well plates and allowed to grow to approximately 70% confluence. Before transfection, DMEM was changed to Opti-MEM I Reduced Serum Medium (Thermo Fisher Scientific). Two µg luciferase reporter plasmid (containing different HPV 33 LCR constructs) and 0.05 µg pRL-TK Control Vector (encoding *Renilla reniformis* luciferase, Promega) were diluted in 250 µl Opti-MEM. Lipofectamine 2000 Reagent (6 µl) was diluted in 250 µl Opti-MEM, and added to the diluted plasmid DNA. After 10 min incubation at room temperature, the mix was added to HeLa cells. After 4 h incubation at 37 °C, the mix was changed to DMEM. Cells were harvested 48 h after transfection by the addition of 250 ml Passive Lysis Buffer (Promega) and one freeze-thaw cycle. The Dual-Luciferase Reporter (DLR) Assay System (Promega) was used to measure the luciferase activity of cell extracts. In the DLR assays, both the firefly luciferase activity and the Renilla luciferase activity of the samples were measured. Renilla luciferase activities were used to standardise for transfection efficiency. All experiments were performed at least three times. Data shown are the means and standard errors of at least 3 independent experiments. The statistical significance of the difference between mean values was evaluated using the ratio t test (a paired t test performed after logarithmic transformation of standardized luciferase values). Statistical significance was accepted at p < 0.05.

### Phylogenetic analysis

The DNA Baser software (Heracle Biosoft, Romania) was used to assemble the HPV 33 LCR sequences. Multiple sequence alignment and construction of phylogenetic trees were performed using MEGA6 software using the maximum likelihood (ML) method [33]. The percentages of replicate trees in which the associated taxa clustered together in the bootstrap test (500 replicates) are shown next to the branches. The sequences of the HPV 33 LCR variants (nt 7116 − 104) reported in this study are deposited in GenBank under the accession numbers OQ725002 - OQ725011.

## Results

The results of sequence analysis of the HPV 33 LCR variants are shown in Table 1. In the 20 HPV 33 positive samples tested, 22 single-nucleotide polymorphisms (SNPs) and a 79 bp long deletion (nt 7596–7674) were demonstrated compared to the prototype sequence. Altogether 10 different variants (designated 33LCRHU01–33LCRHU10) were identified in this study. The A81C nucleotide change was found in all our isolates, but we did not consider it a true SNP, in accordance with previous reports [12, 25, 26]. One of our variants (33LCRHU01), found in 4 samples, had the same LCR sequence as the prototype (M12732), except for the A81C nucleotide change.

The 79 bp deletion and 13 of the 22 SNPs were found in more than one isolates. All of these sequence alterations have been already described in previous reports [13, 16–18, 20, 25]. Nine SNPs were found in single specimens in this study (Table 1). One of them (G7545A) have been described previously [20]. The other 8 unique SNPs were not reported in previous publications, so they were verified by repeated amplification and sequencing of the samples carrying them. Four of our unique SNPs were C > T or G > A nucleotide changes (G7208A, C7397T, C7426T and G7545A), which may be APOBEC3 (apolipoprotein B mRNA editing enzyme catalytic polypeptide 3) signature mutations that have been already described for other HPV types [34, 35].

**Table 1:** Nucleotide sequence alterations in HPV 33 LCR variants

Intratype group	variant ID	7141	7208	7227	7397	7404	7422	7426	7436	7443	7454	7479	7535	7537	7545	7578	7584	7596–7674	7732	7879	7897	6	18	22	81	Frequency
A1	Ref.	T	G	G	C	T	G	C	A	C	G	A	G	C	G	T	G		C	A	C	C	G	T	A	
A1	33LCRHU01																								C	4
	33LCRHU02											C			A										C	1
	33LCRHU03																A				T				C	3
A2	33LCRHU04			A		A	T				A		A	A				DEL	G			G	A		C	2
	33LCRHU05			A	T	A	T				A		A	A				DEL	G			G	A		C	1
	33LCRHU06			A		A	T		C		A		A	A				DEL	G			G	A	C	C	1
	33LCRHU07		A	A		A	T	T			A		A	A				DEL	G			G	A		C	1
	33LCRHU08			A		A	T			T	A		A	A				DEL	G	G		G	A		C	5
	33LCRHU09			A		A	T			T	A		A	A		C		DEL	G	G		G	A		C	1
	33LCRHU10	G		A		A	T			T	A		A	A				DEL	G	G		G	A		C	1

Nucleotide changes found in the different HPV 33 LCR variants at the indicated nucleotide positions compared to the reference sequence (Ref., GenBank accession number M12732). Frequency indicates the number of isolates identified for each variant

DEL: 79 bp deletion

**Fig. 1:**
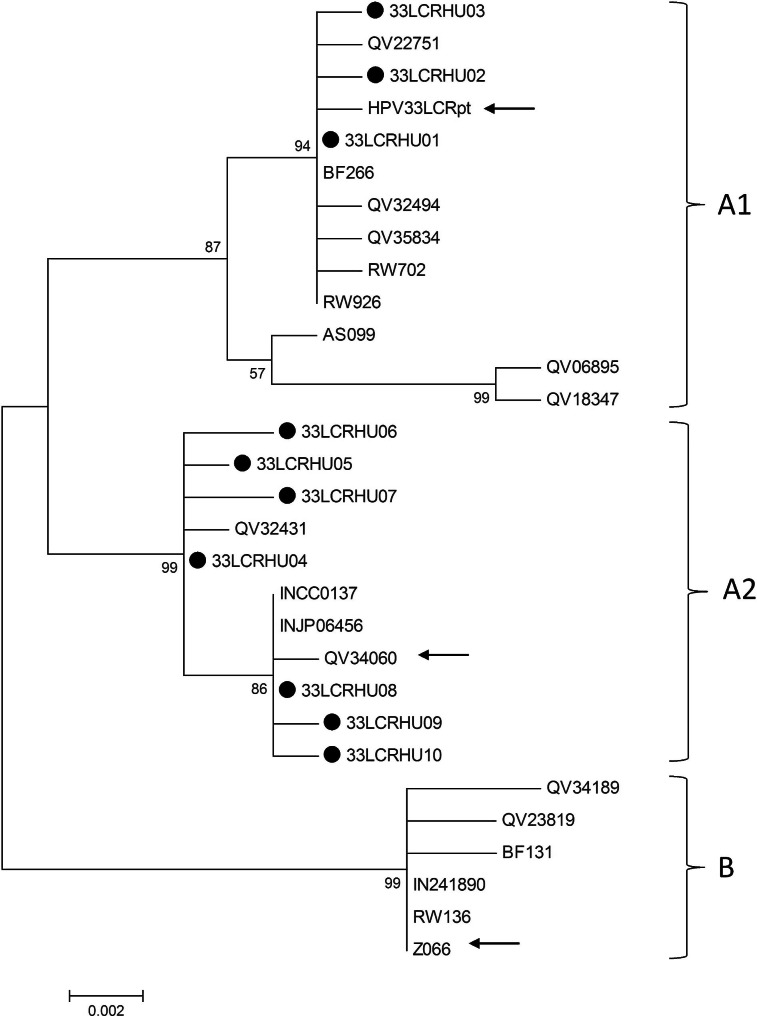
Phylogenetic tree constructed from Hungarian HPV 33 LCR sequences reported in the current study and from overlapping HPV 33 LCR sequences (Chen et al., 2011) from the GenBank database. The bootstrap consensus tree shown was constructed using the maximum likelihood (ML) method. The Hungarian isolates are indicated by black circles. Reference intratype variant isolates are indicated by arrows. Only one isolate is included from each Hungarian variant. Bootstrap values equal to or higher than 57% are shown next to the branches

For the phylogenetic analysis of HPV 33 variants, a maximum likelihood (ML) tree was constructed from our LCR sequences and from whole genome HPV 33 sequences reported in a previous article [13]. As shown in Table 1 and Fig. 1, three of our LCR variants (found in 8 samples) clustered in sublineage A1 of HPV 33. These variants were very similar to the reference HPV 33 sequence, which also belongs to sublineage A1 [13]. The other 7 variants (found in altogether 12 samples) clustered in sublineage A2, along with variants reported previously. Compared to the reference HPV 33 LCR sequence, variants belonging to sublineage A2 had several single-nucleotide changes, and each contained the 79 bp deletion as previously reported [13, 16–18, 20, 25]. In this study, no variants were found that would belong to lineage B, which is characteristic for Africa [12].

**Fig. 2:**
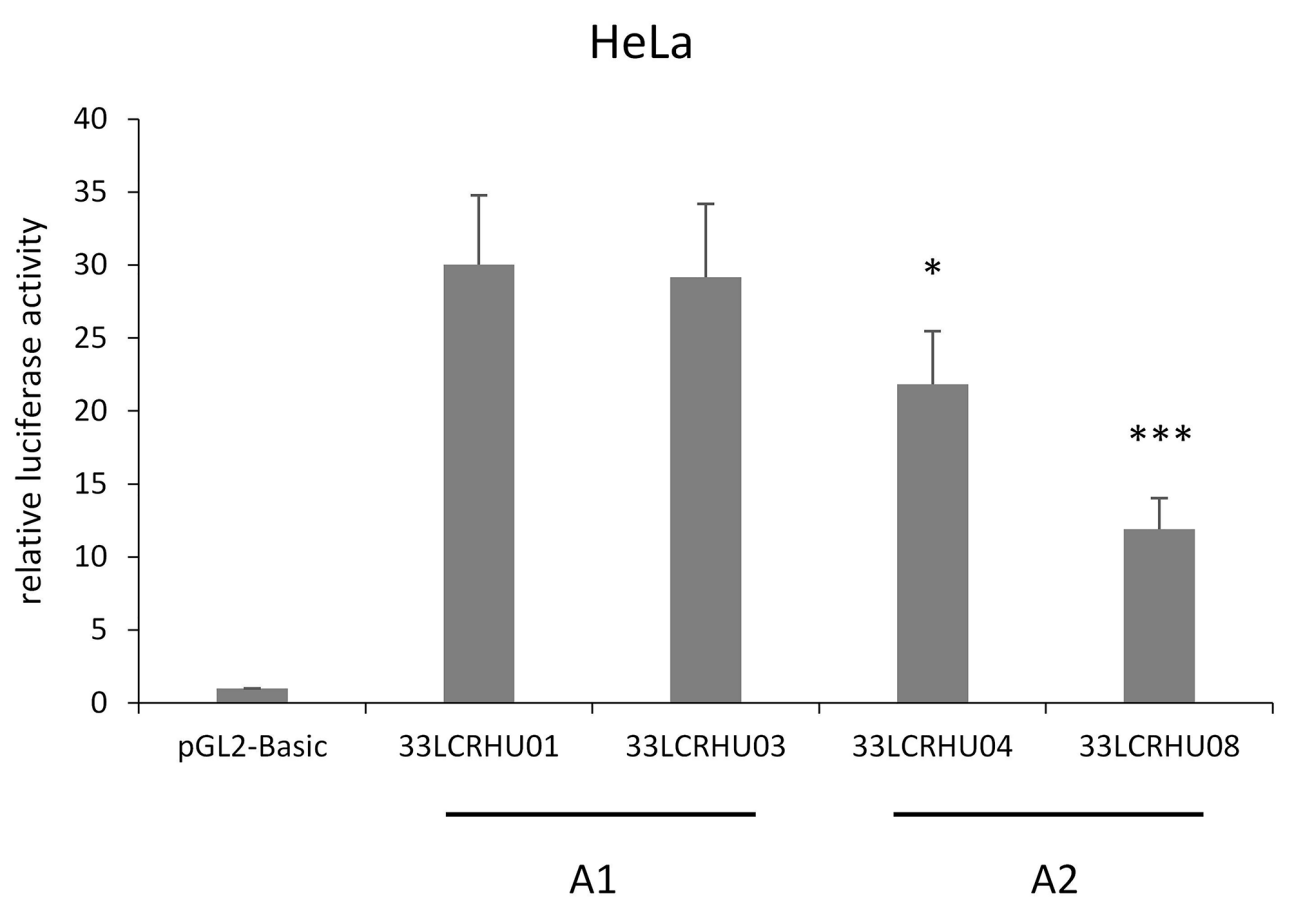
Transcriptional activity of HPV 33 LCR variants in HeLa cells. Cells were co-transfected with luciferase reporter plasmids containing full-length HPV 33 LCR sequence variants and pRL-TK Control Vector. Luciferase activities are shown relative to the activity of cells transfected with pGL2-Basic vector. Data are from four independent experiments, with standard errors shown as error bars. Stars indicate the significance of difference in transcriptional activity compared to 33LCRHU01 transfected cells according to ratio t-test (*p < 0.05, ***p < 0.001). The variant lineages comprising the tested variants are indicated under the bars

As the nucleotide changes found in the HPV 33 LCR variants may involve transcription factor binding sites, we decided to explore if there are differences in the transcriptional activities of the variants. To this end, representative variants (that were found in more than one clinical sample) were cloned into a luciferase reporter vector and analysed in luciferase assays performed in the cervical cancer cell line Hela. Each tested HPV 33 LCR variant had relatively high transcriptional activity compared to the empty reporter vector (pGL2-Basic), and there were significant differences between the transcriptional activities of the variants (Fig. 2). Variants belonging to sublineage A1 (33LCRHU01 and 33LCRHU03) had very similar transcriptional activities, while variants belonging to sublineage A2 (33LCRHU04 and 33LCRHU08) had reduced transcriptional activities compared to variants belonging to sublineage A1. In addition, the 2 variants belonging to sublineage A2 had different transcriptional activities when compared to one another (Fig. 2).

**Fig. 3:**
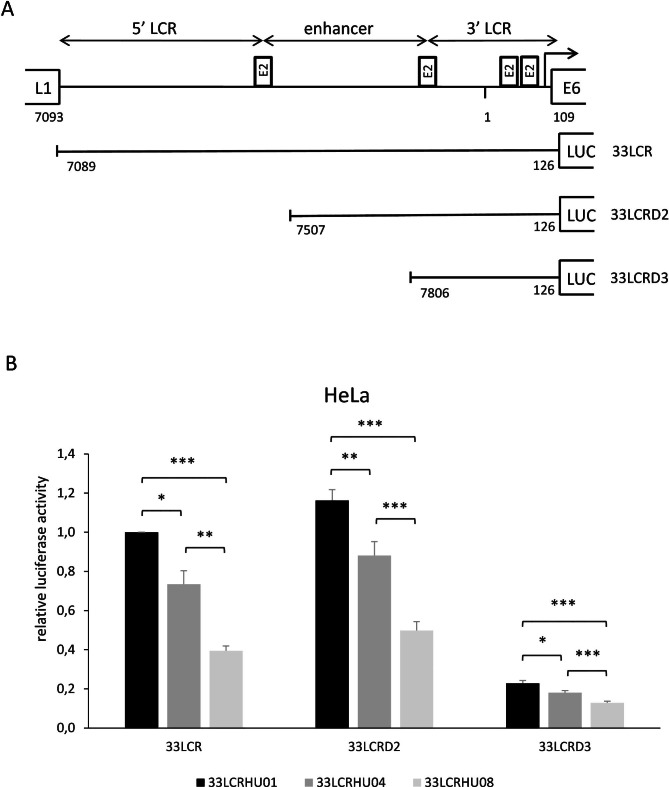
Transcriptional activity of HPV 33 LCR deletion constructs representing different intratype lineages. **A**: Schematic representation of the different LCR fragments of variants cloned in front of the luciferase gene in the reporter vector pGL2-Basic. **B**: Relative luciferase activities of HeLa cells transiently transfected with HPV 33 LCR deletion constructs. Luciferase activities are shown relative to the activity of cells transfected with 33LCRHU01 full-length HPV 33 LCR vector construct. Data are from four independent experiments, with standard errors shown as error bars. Statistical analysis was performed using ratio t test. Stars indicate the significance of difference in transcriptional activity compared to 33LCRHU01 or 33LCRHU04 transfected cells within each group of bars as indicated (*p < 0.05, **p < = 0.005, ***p < 0.001)

The LCR of genital HPV types can be divided into 3 different functional regions [7]. The 5’ LCR (the region between the L1 stop codon and the promoter-distal E2 binding site) contains the late polyadenylation signal. The central region contains the epithelial-specific enhancer, while the 3’ LCR contains the origin of replication and the promoter of the early genes (Fig. 3A). To explore which of these functional regions (and the nucleotide changes found in them) may be responsible for the different transcriptional activities of the LCR variants, reporter constructs containing different segments of some representative HPV 33 LCR variants were tested in luciferase assays (Fig. 3B). Our results showed that the 5’ LCR did not contribute to the transcriptional activities of the different HPV 33 LCR variants, as the transcriptional activities of the 33LCRD2 constructs (lacking the 5’ LCR) were not lower than that of the corresponding full-length constructs. On the other hand, the differences found between the different variants in the 33LCRD2 constructs were very similar to those found in the full length LCR constructs. For example, the 33LCRHU08 variant had significantly reduced transcriptional activity compared to the 33LCRHU04 variant when tested using the 33LCRD2 constructs (Fig. 3B). The single difference between the two constructs was the A7879G nucleotide change (Table 1), proving that this particular nucleotide change has a significant inhibitory effect on the transcriptional activity of the HPV 33 LCR in Hela cells.

The 33LCRD3 constructs had highly reduced activities compared to the full length constructs or to the 33LCRD2 constructs, as they contained only the basic promoter but not the central enhancer region (Fig. 3B). However, there were still significant differences in the transcriptional activities of the different variants also in the case of the 33LCRD3 constructs. This suggests that the nucleotide changes found in the 3’ LCR region (that is contained in the 33LCRD3 constructs) also contribute to the differences in the transcriptional activities of the tested HPV 33 LCR variants. Also in the case of the 33LCRD3 constructs, the transcriptional activity of the 33LCRHU08 variant was significantly reduced compared to that of the 33LCRHU04 variant. The single difference between the 2 constructs was again the A7879G variation (Table 1), showing that this nucleotide change is responsible for the different transcriptional activities of the 2 variants belonging to sublineage A2.

## Discussion

Intratype variants belonging to different HPV 33 (sub)lineages were reported to have differences in clinical behaviour. Specifically, sublineage A1 was found to have higher oncogenic potential than sublineage A2 or lineage B [12, 21–23]. Differences in the clinical behaviour might be explained by differences in some biological activities of HPV 33 variants that can be explored by *in silico* or in vitro functional studies. For example, amino acid changes in the E6 and E7 proteins of HPV 33 variants were reported to modify T-cell epitopes, which might have an effect on the cellular immune response against the E6/E7 protein variants [31]. Furthermore, differences were found between different HPV 33 E6 variants in the ability to induce the degradation of cellular p53 and MAGI-3 proteins [24]. However, functional differences between intratype variants might be also caused by differences in the expression level of the viral oncogenes. As the expression of the E6/E7 oncogenes of high-risk HPVs is governed by the LCR, intratype variants with different transcriptional activities of the LCR might have also different oncogenic potential.

In the case of HPV 16, which is the most prevalent and most studied genotype, relatively good correlation was found between the transcriptional activity of the LCR and the oncogenic potential of the variants [36–39]. The intratype variants were found to have different transcriptional activities also in other high-risk genotypes, such as HPV 18, 31, 33 and 58 [25, 26, 40–45]. As far as HPV 33 is concerned, LCR variants belonging to the sublineage A1 were reported to have higher transcriptional activities than those belonging to sublineage A2 or lineage B in primary human keratinocytes [25].

The goal of the present study was to characterise HPV 33 LCR variants that can be isolated in Hungary, and to see if there are differences in the transcriptional activities of the variants. The results of phylogenetic analysis showed that, out of the 20 HPV 33 positive samples analysed in this study, 8 samples (representing 3 different variants) belonged to sublineage A1. Twelve samples (representing 7 different variants) were found to belong to sublineage A2. We did not have any isolates belonging to the (sub)lineages A3, B or C which are not characteristic in Europe [12].

The 79 bp deletion, which was found in each isolate belonging to sublineage A2 in this study, has been already described in previous papers [13, 16–18, 20]. In fact, this deletion is rather a lack of duplication of a 79 bp sequence. This duplication is characteristic for the variants belonging to sublineage A1, but is not found in other sublineages. However, as the sequence differences in HPV variants are officially compared to the prototype sequence (belonging to sublineage A1 by definition), this particular sequence change is described as a deletion also in this paper.

In addition to the 79 bp deletion, most of the SNPs found in HPV 33 LCR in our samples have been previously described [13, 16–18, 20]. Eight SNPs found in this study in single specimens have not yet been reported. These novel SNPs are most probably real nucleotide changes in the samples as they were validated by repeated amplification and sequencing. Four of our unique SNPs were C > T or G > A nucleotide changes. These mutations could have been induced by the cellular cytidine deaminase enzyme APOBEC3 as described previously for HPV types 1 and 16 [34, 35].

The transcriptional activities of some representative HPV 33 LCR variants were tested in luciferase reporter assays performed after transient transfection of HeLa cells. Our results obtained with the full-length reporter constructs showed that variants belonging to sublineage A1 tended to have higher transcriptional activities than those belonging to sublineage A2. Since sublineage A1 was found to have higher oncogenic potential than sublineage A2 [12, 21–23], it is tempting to speculate that this difference in oncogenicity might be at least partially caused by the differences in the transcriptional activities of the variants. Our results also showed that there may be differences in the transcriptional activities of variants belonging to the same sublineage. Specifically, the 33LCRHU08 variant had significantly reduced transcriptional activity when compared to the 33LCRHU04 variant, although both variants belonged to sublineage A2.

In an effort to localise the regions of the HPV 33 LCR that are responsible for the differences in the transcriptional activities of the variants, deletion constructs were prepared from 3 representative variants and tested in luciferase reporter assays. Our results showed that the 5’ LCR (the region between the L1 stop codon and the promoter-distal E2 binding site) had no significant enhancer activity, and the nucleotide changes found here are probably not responsible for the different transcriptional activities of the variants. It should be noted that this segment of HPV 33 LCR was recently reported to contain an ORF (open reading frame) potentially encoding a 116-amino acid protein [32].

It is not easy to identify precisely the nucleotide changes that are responsible for the different transcriptional activities of the variants belonging to the different sublineages (such as 33LCRHU01 belonging to A1 and 33LCRHU04 belonging to A2). It is probable that the changes found in the central enhancer region of the LCR (such as the 79-bp deletion and the nucleotide changes C7537A and C7732G) are at least partially responsible for the functional differences as suggested by Alvarez and co-workers [25]. On the other hand, our results obtained with the D3 deletion constructs suggest that the nucleotide changes found in the 3’ LCR segment (such as C6G and G18A) also contribute to the differences in the transcriptional activities of lineage A1 and A2 variants.

The genetic change that is responsible for the different transcriptional activities of the two variants belonging to sublineage A2 (33LCRHU04 and 33LCRHU08) could be easily identified. Our results obtained with the D2 and D3 deletion constructs clearly showed that this difference is caused by the A7879G variation. This finding is in accordance with the results of Alvarez and co-workers showing the repressive effect of the A7879G change on the activity of the HPV 33 early promoter in Hela cells [25].

A limitation of this study was that we could not perform a phylogenetic and functional analysis of HPV 33 variants belonging to (sub)lineages that are not present in Europe. On the other hand, due to the small number of samples we could not analyse the clinical behaviour of the variants. Therefore, further clinical and experimental studies are warranted to prove the correlation between intratype variation and the oncogenic potential of HPV 33.

## Conclusions

The reporter assays performed in this study showed that the HPV 33 LCR variants belonging to sublineage A2 have reduced transcriptional activities compared to those belonging to sublineage A1. This finding is in accordance with clinical data showing that sublineage A1 has higher oncogenic potential than sublineage A2. Our results also showed that variants belonging to the same sublineage may have significantly different transcriptional activities. The different transcriptional activities of the 2 variants belonging to sublineage A2 was shown here to be caused by a nucleotide variation in the 3’ LCR segment (A7879G). Our results can help to understand the correlation between LCR polymorphism and the oncogenic potential of HPV 33 variants.

## Data Availability

All datasets analysed in this study are available from the corresponding author on reasonable request.
